# Supplementary material for: Preparation and Evaluation of Antidiabetic Agents of Berberine Organic Acid Salts for Enhancing the Bioavailability
Source: Molecules. 2018 Dec 28;24(1):103. doi: 10.3390/molecules24010103 (PMC6337101; doi:10.3390/molecules24010103)
Supplement: Supplementary file 1 [file molecules-24-00103-s001.pdf]

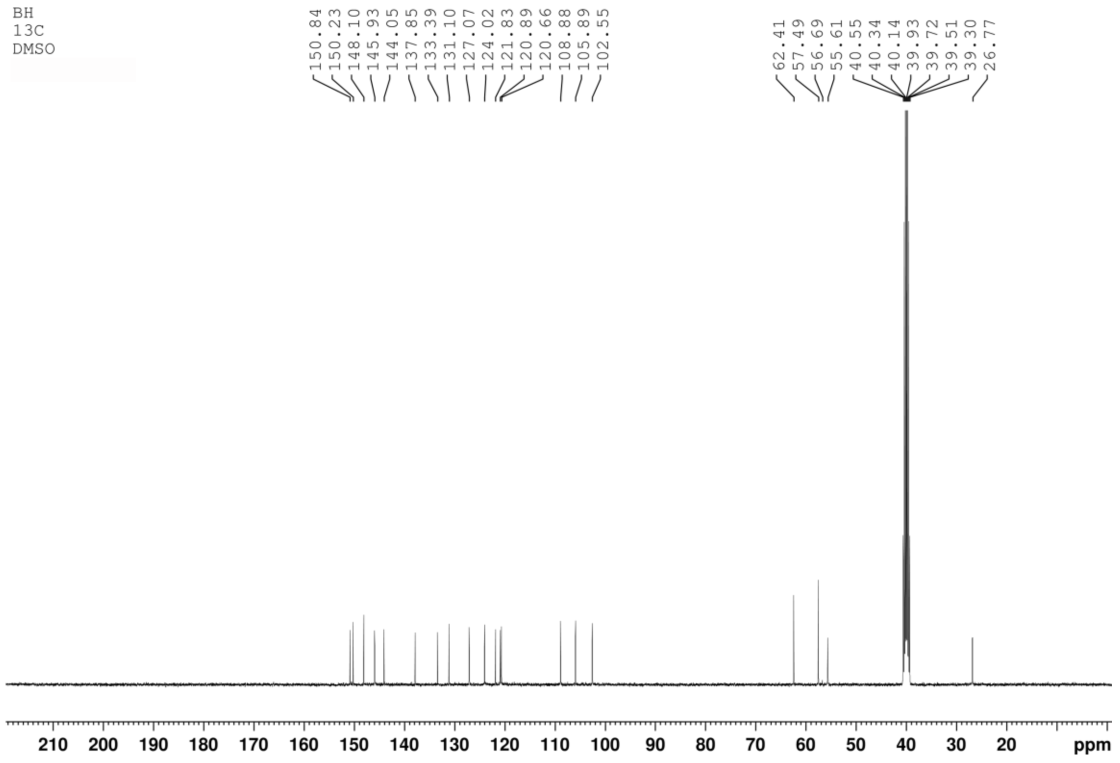

BH
13C
DMSO
150.84
150.23
148.10
145.93
144.05
137.85
133.39
131.10
127.07
124.02
121.83
120.89
120.66
108.88
105.89
102.55
62.41
57.49
56.69
55.61
40.55
40.34
40.14
39.93
39.72
39.51
39.30
26.77
210 200 190 180 170 160 150 140 130 120 110 100 90 80 70 60 50 40 30 20 ppm

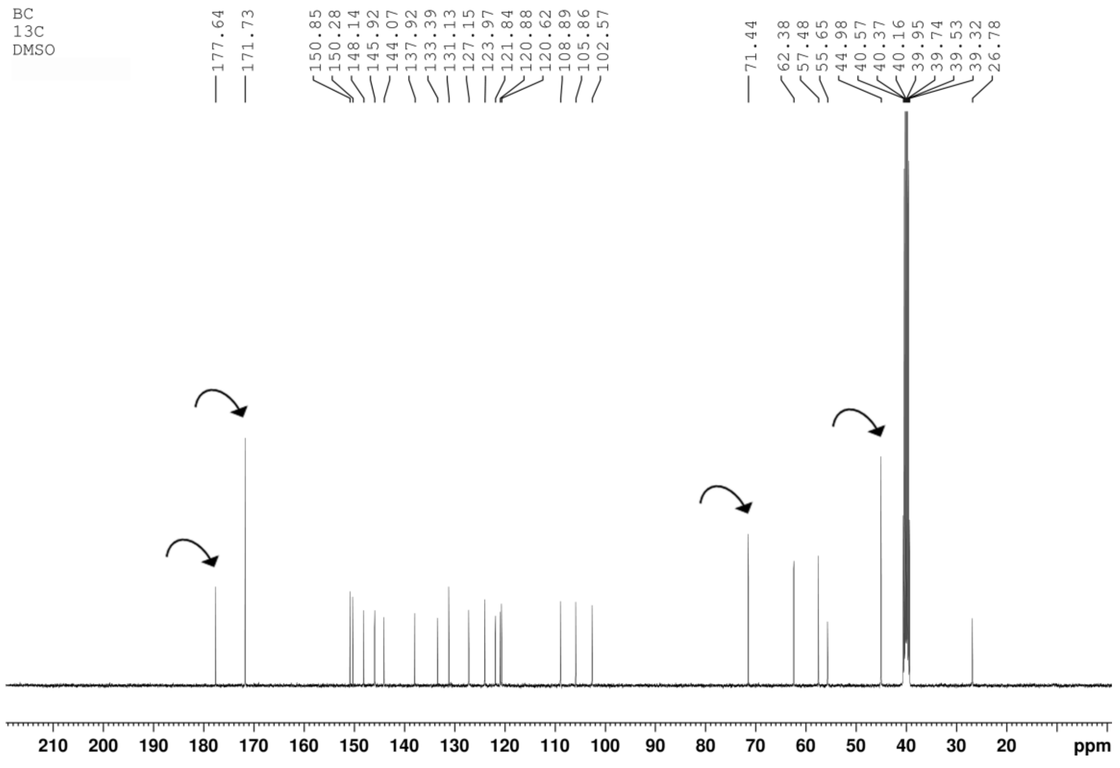

BC
13C
DMSO
177.64
171.73
150.85
150.28
148.14
145.92
144.07
137.92
133.39
131.13
127.15
123.97
121.84
120.88
120.62
108.89
105.86
102.57
71.44
62.38
57.48
55.65
44.98
40.57
40.37
40.16
39.95
39.74
39.53
39.32
26.78
210 200 190 180 170 160 150 140 130 120 110 100 90 80 70 60 50 40 30 20 ppm

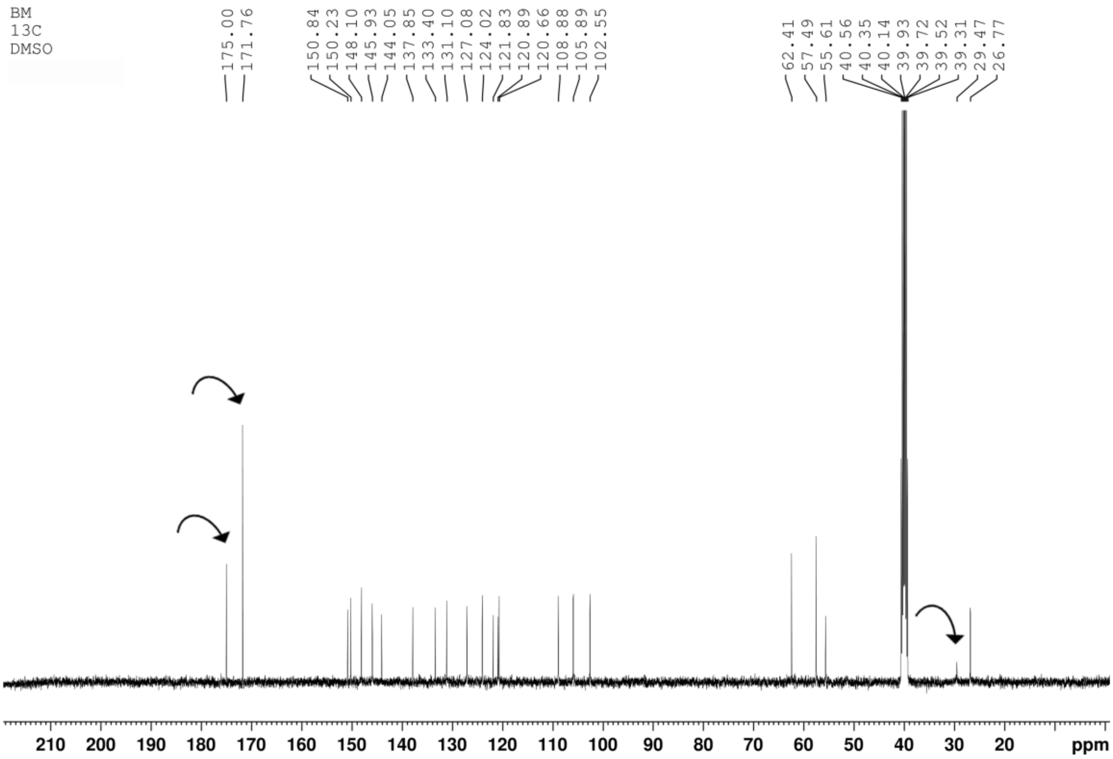
BM
13C
DMSO
175.00
171.76
150.84
150.23
148.10
145.93
144.05
137.85
133.40
131.10
127.08
124.02
121.83
120.89
120.66
108.88
105.89
102.55
62.41
57.49
55.61
40.56
40.35
40.14
39.93
39.72
39.52
39.31
29.47
26.77
210 200 190 180 170 160 150 140 130 120 110 100 90 80 70 60 50 40 30 20 ppm

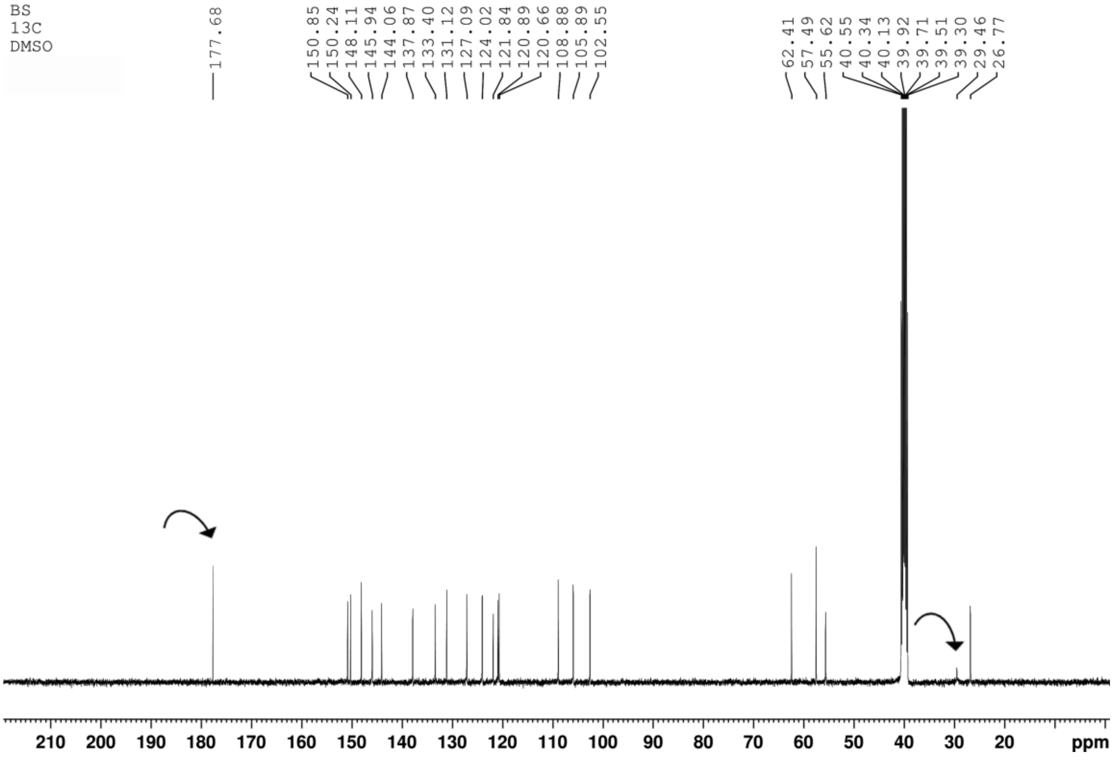
BS
13C
DMSO
177.68
150.85
150.24
148.11
145.94
144.06
137.87
133.40
131.12
127.09
124.02
121.84
120.89
120.66
108.88
105.89
102.55
62.41
57.49
55.62
40.55
40.34
40.13
39.92
39.71
39.51
39.30
29.46
26.77
210 200 190 180 170 160 150 140 130 120 110 100 90 80 70 60 50 40 30 20 ppm

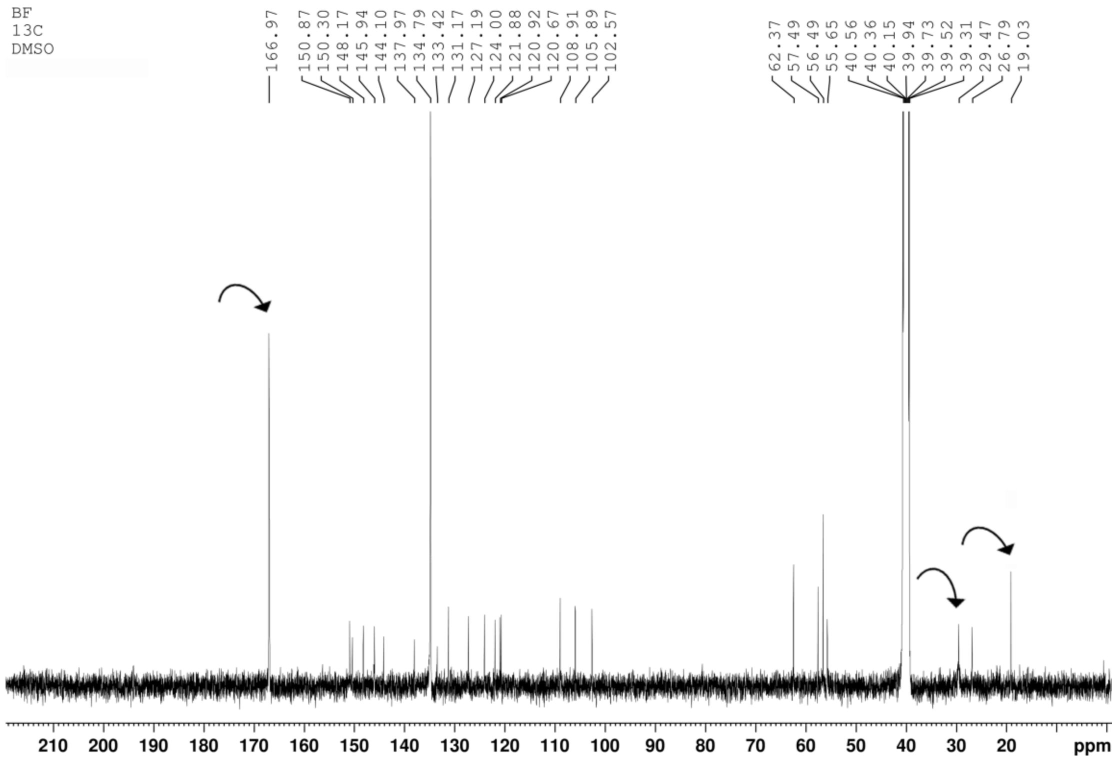

Fig. A.1. ^{13}CNMR spectra of berberine hydrochloride (BH), berberine citrate (BC), berberine succinate (BS), berberine malate (BM) and berberine fumarate (BF). (100MHz, in DMSO). The arrow in the picture refers to organic acid carbon.
